# Proteomic changes in the hippocampus of large mammals after total-body low dose radiation

**DOI:** 10.1371/journal.pone.0296903

**Published:** 2024-03-01

**Authors:** Diego Iacono, Kathleen Hatch, Erin K. Murphy, Jeremy Post, Robert N. Cole, Daniel P. Perl, Regina M. Day

**Affiliations:** 1 DoD/USU Brain Tissue Repository & Neuropathology Program, Uniformed Services University (USU), Bethesda, Maryland, United States of America; 2 Department of Neurology, F. Edward Hébert School of Medicine, Uniformed Services University (USU), Bethesda, Maryland, United States of America; 3 Department of Pathology, F. Edward Hébert School of Medicine, Uniformed Services University (USU), Bethesda, Maryland, United States of America; 4 Neuroscience Program, Department of Anatomy, Physiology and Genetics (APG), F. Edward Hébert School of Medicine, Uniformed Services University (USU), Bethesda, Maryland, United States of America; 5 The Henry M. Jackson Foundation for the Advancement of Military Medicine, Inc. (HJF), Bethesda, Maryland, United States of America; 6 Neurodegeneration Disorders Clinic, National Institute of Neurological Disorders and Stroke, NINDS, NIH, Bethesda, Maryland, United States of America; 7 Mass Spectrometry and Proteomics, Department of Biological Chemistry, Johns Hopkins University, School of Medicine, Baltimore, Maryland, United States of America; 8 Department of Pharmacology and Molecular Therapeutics, Uniformed Services University (USU), Bethesda, Maryland, United States of America; University of Florida, UNITED STATES

## Abstract

There is a growing interest in low dose radiation (LDR) to counteract neurodegeneration. However, LDR effects on normal brain have not been completely explored yet. Recent analyses showed that LDR exposure to normal brain tissue causes expression level changes of different proteins including neurodegeneration-associated proteins. We assessed the proteomic changes occurring in radiated vs. sham normal swine brains. Due to its involvement in various neurodegenerative processes, including those associated with cognitive changes after high dose radiation exposure, we focused on the hippocampus first. We observed significant proteomic changes in the hippocampus of radiated vs. sham swine after LDR (1.79Gy). Mass spectrometry results showed 190 up-regulated and 120 down-regulated proteins after LDR. Western blotting analyses confirmed increased levels of *TPM1*, *TPM4*, *PCP4* and *NPY* (all proteins decreased in various neurodegenerative processes, with *NPY* and *PCP4* known to be neuroprotective) in radiated vs. sham swine. These data support the use of LDR as a potential beneficial tool to interfere with neurodegenerative processes and perhaps other brain-related disorders, including behavioral disorders.

## Introduction

Radiation exposures, at different total amounts and rates, are routinely used for various medical purposes (e.g. X-ray imaging, tumor treatments, diagnostic tests, etc.). However, high dose radiation (HDR) exposure to the central nervous system (CNS), unless therapeutically indicated, is generally avoided due to the possibility of major neurological consequences [1, 2]. Aside from specific therapeutic interventions and natural radiation background exposure, the CNS, and the brain in particular, may be exposed to different amounts and rates of ionizing radiation through various circumstances such as occupational activities, environmental hazards, nuclear power accidents, nuclear war, and space traveling [3–5]. Surprisingly, though, the exact molecular and pathological effects of radiation (i.e., γ-radiation), and low dose radiation (LDR) in particular, on normal brain tissue are not completely known or even explored [6, 7].

In recent years, there has been a new and growing interest in the potential use of LDR as a therapeutic intervention against brain disorders, and in particular, neurodegenerative disorders [8]. New studies have started to provide initial and robust evidence that seems to support the possible applicability of LDR as a viable and beneficial intervention for neurodegenerative disorders such as Alzheimer’s Disease (AD)—currently the most frequent form of dementia among elders—or Parkinson’s disease (PD), the most frequent movement disorder across human populations [9–12]. Moreover, during the last few decades of neurobiological research, Mass Spectrometry (MS)-based proteomic studies have used brain tissues, either from animal or human specimens, and identified a large number of proteins and protein expression level changes associated with neurological conditions such as AD, PD, Fronto-temporal dementia (FTD), amyotrophic lateral sclerosis (ALS), multiple sclerosis (MS), traumatic brain injury (TBI) and other brain-related disorders, including psychiatric disorders [13–17]. One of the primary advantages of the MS-based proteomic approach is the unbiased identification of protein abundance changes. The MS-proteomic approach provides unique results irrespective of any preconceived interpretation or hypothesis [18]. This MS-related methodological strength is crucial when studying the myriad of possible and simultaneous neurobiological processes undergoing molecular changes in the brain following pathological events, especially environmental events, such as those following radiation exposure, and γ-radiation exposure in particular.

Previous proteomic investigations have been achieved employing various radiation experimental constructs, including chronic low-dose-rate ionizing radiation and cosmic radiation, in order to detect protein expression level changes across different tissues and organs, including the brain [19–21]. However, to the best of our knowledge, no studies have ever reported data on the specific proteomic profile changes caused by a single LDR (sLDR) total-body exposure to the normal hippocampus (Hip) of larger mammals (swine) assessed by MS-proteomic analyses and followed by confirmatory Western Blotting (WB) measurements for the MS-identified proteins.

New molecular and neuropathological data from normal swine exposed to total-body sLDR demonstrated that, at 28 days after irradiation, significant expression level changes of several proteins had occurred in different brain regions of radiated (RAD) vs. non-radiated (sham/SH) swine [22]. Among those proteins showing sLDR-induced expression level changes, hyperphosphorylated-tau protein (pTau) was of particular interest due to its major involvement in the pathomechanisms of AD and other neurodegenerative disorders [23].

Based on those previous findings, we investigated if the MS-based proteomic approach could identify a wider spectrum of brain-protein expression level changes as a direct outcome of a sLDR total-body exposure. Initially, we performed MS-proteomic analyses focusing on the hippocampus region (Hip). The reason to focus on the Hip first consisted in its major and frequent involvement in highly complex cognitive and non-cognitive processes that are often more affected during the natural progression of several neurodegenerative disorders [24]. Furthermore, the Hip is actively involved in multiple physiological and developmental mechanisms including synaptic plasticity, neurogenesis, learning and memory skills acquisition [25]. All these mechanisms could be affected by either natural or man-made higher levels of radiation and could initiate a vast series of long-term effects. This last aspect is actually of special relevance for future neurodevelopmental therapies and their effects as well as in terms of long-term adaptive responses to space travel [26]. Furthermore, the Hip and its subregions have been hypothesized to be involved in those specific pathogenetic processes related to the brain’s responses after cranial radiation exposure in the context of pediatric neuro-oncology radiotherapy procedures [27, 28].

## Results

**Fig 1:**
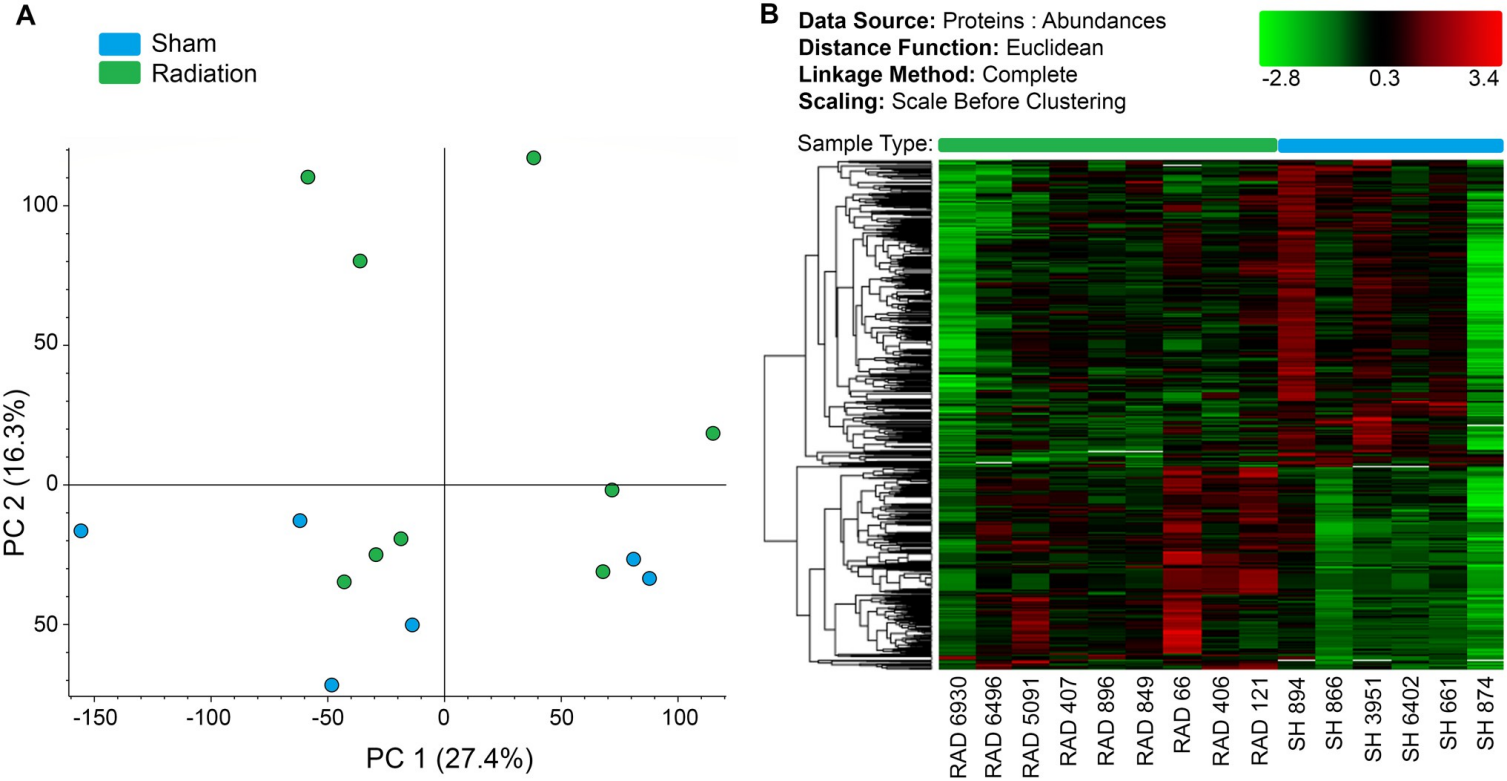
Differential clustering of RAD vs. SH swine. (A) PCA of proteomic profiles shows distinct differential clustering of RAD (green) vs. SH (blue) hippocampal protein expression. (B) Heat map of expression pattern clustering, where green indicates low and red indicates high differential protein abundance. Hierarchal clustering of proteins reveals group-specific abundance trends (see brackets on heat map). Z-score transformation of normalized protein abundances from a quantitative proteomics analysis using isobaric mass tags was applied before performing the hierarchical clustering based on Euclidean distance and complete (furthest neighbors) linkage. The horizontal dendogram shows the proteins in samples that clustered together. RAD n = 9, SH n = 6.

Protein abundance across all samples for proteomic profiling showed optimum quality and consistency for reliable MS-based proteomic analysis (see S1 Fig). The unsupervised Principle Component Analysis (PCA) graph (Fig 1A) demonstrates distinct separation between the RAD (n = 9) and SH (n = 6) Hip groups, with only a few RAD samples approaching SH in the lower half; the heat map (Fig 1B) confirmed this separation, with only one RAD sample appearing within the SH group.

### MS proteomic profiling

**Fig 2:**
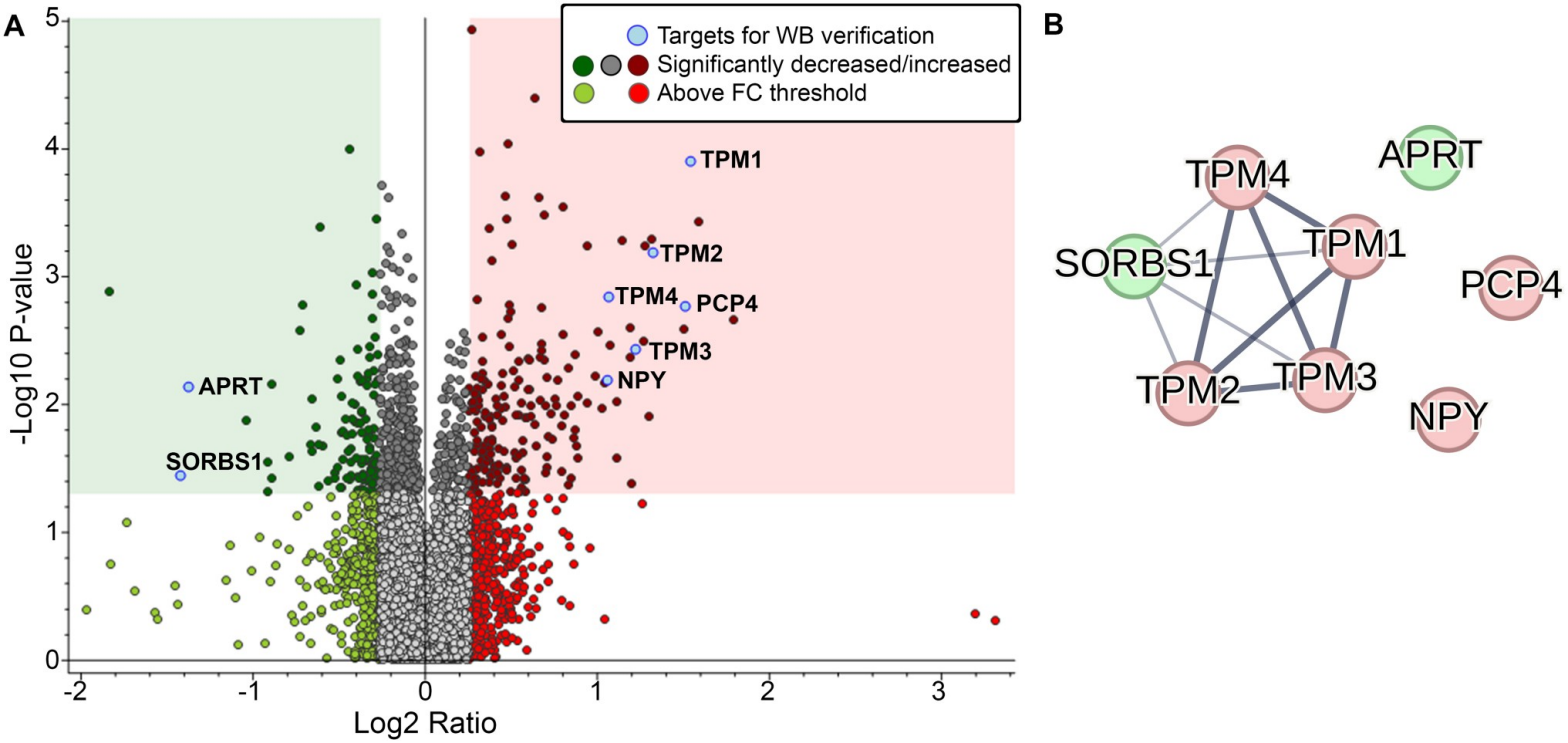
Hippocampal proteomic profiling of RAD vs. SH swine. (A) Volcano plot of log2(FC) vs -log10(p-value) of hippocampal proteomic profiles, with up-regulated proteins on the right (red) and down-regulated proteins on the left (green). Significance threshold of *p*<0.05 is indicated by shading, with all significantly changed proteins with log2(FC)≥0.26 included in our analysis shown in the corresponding color shaded boxes (*p*-value of per group ratio calculated by *t*-test; fold changes visualized as log2 of abundance ratio). We identified 190 up-regulated (red square) and 120 down-regulated (green square) proteins within these criteria through the proteomic profiling. Location of target proteins selected for WB verification are indicated by blue dots on the volcano plot (A), and the STRING interaction network of these proteins is illustrated (B).

MS-proteomic analysis identified 8,325 protein groups, with 7,226 quantified, from 57,503 peptide groups sequenced from 126,195 peptide-spectral matches at 5% FDR. (see full list of identified proteins in S1 Table). Based on our thresholding parameters (*p*<0.05; Log2 FC = 0.26), a total of 190 proteins were increased and 120 proteins were decreased in abundance in the Hip of RAD vs. SH swine (see list for up- and down-regulated proteins in S2 Table). A volcano plot of the total increased (red square) and decreased (green square) proteins within our threshold parameters in RAD vs. SH hippocampi samples is shown in Fig 2a. The TMT-MS raw data and analysis files have been uploaded to the publicly accessible repository MassIVE and can be found here: https://doi.org/10.25345/C5NZ8113D.

### WB-verified proteomic findings

**Fig 3:**
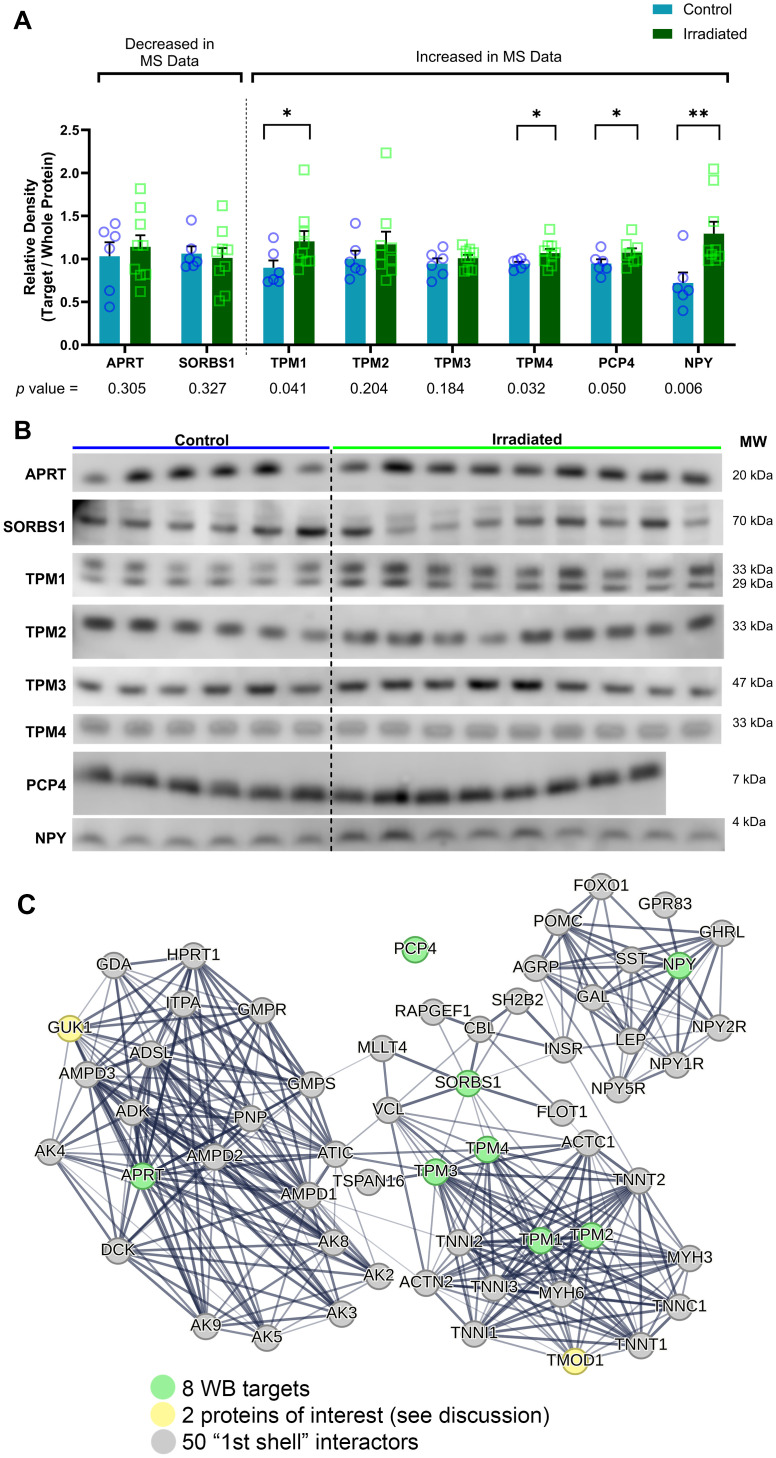
TPM1, TPM4, PCP4 and NPY are identified as having significantly increased abundance in the hippocampus after LDR exposure. (A, B) Western blot verifies the significantly increased abundance in the swine hippocampus in RAD vs SH groups (*p*<0.05; unpaired Student’s *t*-test, one-tailed; RAD n = 9 (n = 8 for PCP4), SH n = 6) initially identified through proteomic analysis. STRING software analyses of the top 50 (C) protein interactions with the eight WB targets identify potential mechanisms of involvement in the brain’s response to LDR.

Based on our broader thresholds to identify differentially abundant proteins among the proteomic analysis, we narrowed the selection criteria to proteins with a Log2 FC>±1 to identify targets with a high likelihood of congruent verification by WB analyses. Based on these criteria, we found significant increase of *Tropomyosin 1* (*TPM1)*, *Tropomyosin 4 (TPM4)*, *Purkinje Cell Protein 4 (PCP4*, *a*.*k*.*a*. *PEP19)* and *Neuropeptide Y (NPY)* in RAD vs. SH groups. We did not find a significant increase through WB in abundance of *Tropomyosin 2 (TPM2)* and *Tropomyosin 3 (TPM3)*; and no significant decrease in abundance of *Adenine Phosphoribosyltransferase* (*APRT)* or *Sorbin and SH3 domain containing 1 (SORBS1)* was measured. See WB quantitative outcomes in Fig 3a (please see S2 Fig for raw WB images, and Fig 3b for representative WB strips).

### STRING analysis

**Fig 4:**
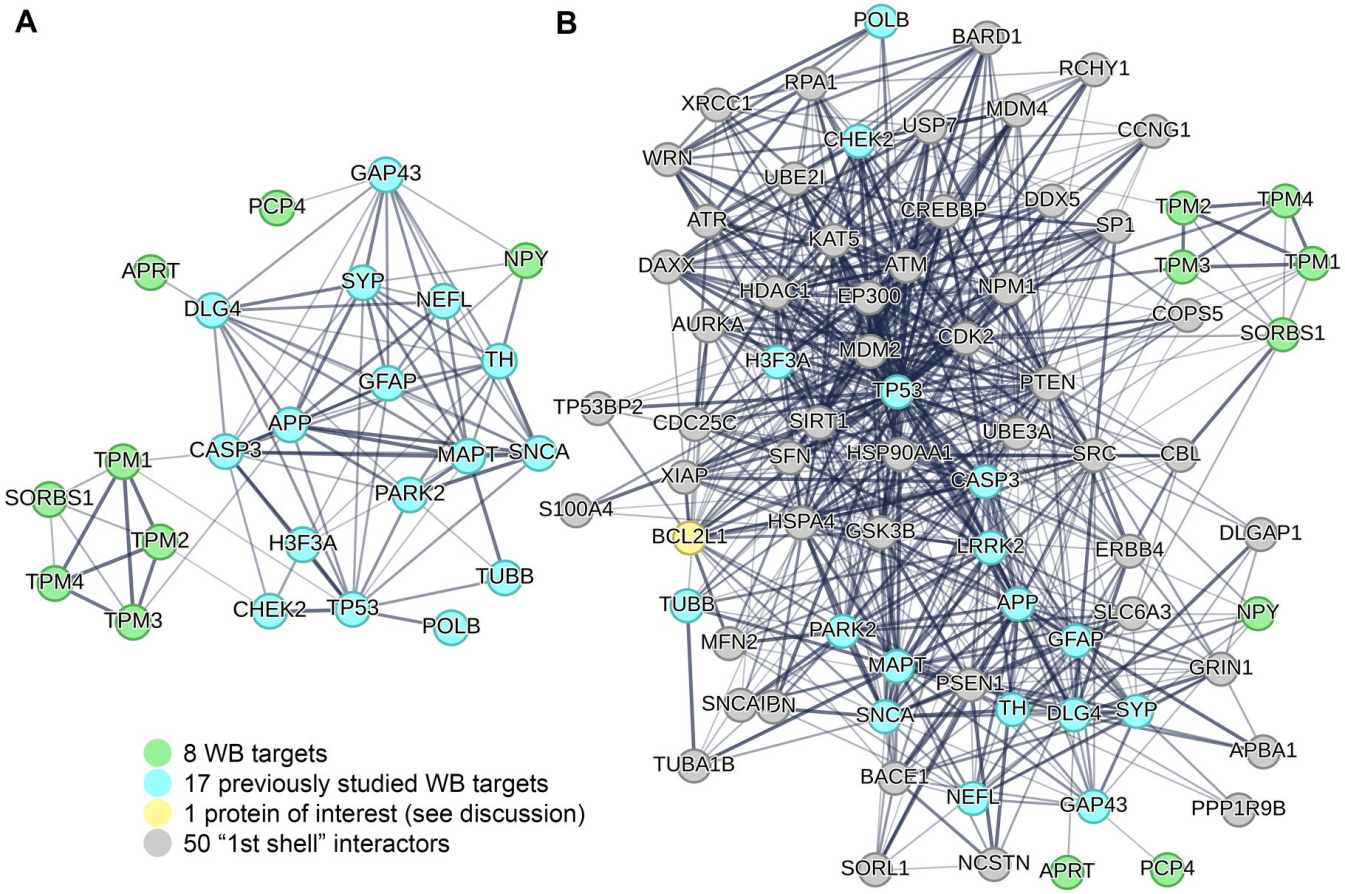
Fitting selected WB targets into the broader network of neurodegenerative protein interactions. (A) STRING analysis of protein interactions following input of eight target proteins and 18 previously investigated proteins involved in neurodegeneration after LDR (MAPT, SNCA, PARK2, LRRK2, APP, TUBB, TH, SYP, CASP3, NEFL, GFAP, GAP43, DLG4, PPP1R9B, TP53, CHEK2, H3F3A and POLB) (22), demonstrating possible interactions of our target proteins within this wider neurodegenerative network. The STRING interaction network with 50 closest interactors is also visualized (B).

The STRING database (https://string-db.org/) is a powerful tool that uses data-mining to provide information regarding protein-protein interactions (known physical interactions and functional associations based on scientific literature, computational predictions from co-expression, genomic content, and experimental data), as well as offering network visualization of the strength of those interactions and functional enrichment analyses [30]. Using our eight WB protein targets as input against a *Homo sapiens* background, we found that several of the proteins selected for verification are actually connected, and more specifically those associated with cytoskeletal function (*TPM1*, *TPM2*, *TPM3*, and *TPM4*) (Fig 2B). To explore potential associated protein interactions with the target proteins, STRING was used to populate the network with 50 1^st^ shell interactor proteins (Fig 3C). Based on the STRING enrichment analysis of this network, it confirms that the target proteins and close interactors are predominantly involved in actin binding and cytoskeletal structure, purine metabolism and salvage, and cell signaling (see S3 Table). Of the protein interactors in this network, *Guanylate Kinase 1* (*GUK1)* is also found to be significantly decreased in RAD vs SH Hip samples. To understand how these target proteins interact with neurodegenerative-associated proteins, we input to STRING the eight WB target proteins as well as 18 previously investigated proteins (*MAPT*, *SNCA*, *PARK2*, *LRRK2*, *APP*, *TUBB*, *TH*, *SYP*, *CASP3*, *NEFL*, *GFAP*, *GAP43*, *DLG4*, *PPP1R9B*, *TP53*, *CHEK2*, *H3F3A* and *POLB*) (Fig 4a) [22]. Not only did these analyses reveal interactions between these eight novel targets and the wider neurodegenerative network, but it also uncovered possible involvement of these protein networks in opioid receptor signaling and dopamine binding (Fig 4a and 4b). Specifically, within this neurodegenerative-focused interaction network, *BCL2 like 1 (BCL2L1)* is found to be significantly increased in RAD vs SH Hip (see S4 Table for STRING enrichment analysis).

### DAVID analysis

**Fig 5:**
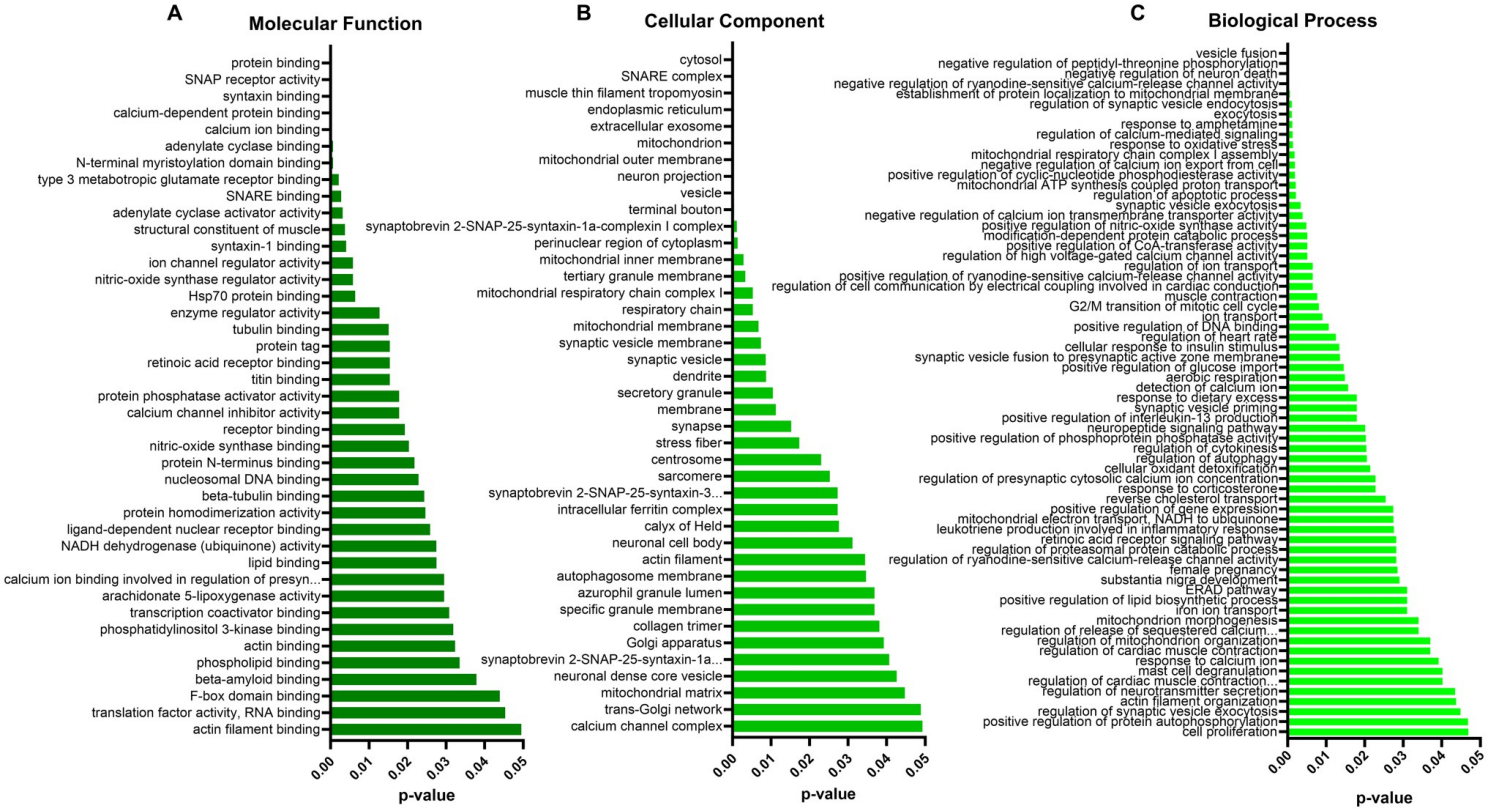
DAVID analyses of differentially abundant hippocampal proteins identified through proteomic analysis of RAD vs. SH swine. Following input of a condensed list of 310 proteins identified through proteomic analysis as having differential abundance in the hippocampus after LDR, DAVID enrichment analysis of GO relationships revealed 41 molecular functions (A), 41 cellular components (B) and 68 biological processes (C) as potentially significantly affected.

The Database for Annotation, Visualization and Integrated Discovery (DAVID) is another powerful tool used to understand biological meaning behind large gene datasets through functional annotation, built on a vast knowledgebase derived from multiple sources (https://david.ncifcrf.gov/) [31, 32]. After the list of 310 differentially abundant proteins following sLDR was input to DAVID for Gene Ontology (GO) analyses against a *Homo sapiens* background, 291 were identified with official gene symbols. Using DAVID’s functional annotation tool, we selected the GO analyses for molecular function (96.9% coverage, includes 282/291 terms from our list), cellular component (97.6% coverage, 284/291 terms) and biological process (92.8% coverage, 270/291 terms). This analysis identified 41 significantly (*p*<0.05) enriched molecular functions (Fig 5a), 41 significantly enriched cellular components (Fig 5b), and 68 significantly enriched biological processes (Fig 5c). The GO results indicate a possible impact of sLDR on calcium binding and activity, beta-amyloid binding and nucleosomal DNA binding (molecular functions), mitochondrial components, synapses, dendrites and neuronal projections, and SNARE complex (cellular components), as well as mitochondrial activity, vesicle function, response to oxidative stress, negative regulation of neuron death and regulation of apoptosis (biological processes) (see S5 Table for DAVID GO enrichment analysis).

## Discussion

MS-based proteomic methodology is a powerful instrument to deliver large quantitative datasets to measure differences between different normal or pathological conditions without the influence of previously formulated hypotheses. Accordingly, the MS-proteomic approach (and MS-TMT method in particular) has the impressive potential to provide novel and often unexpected findings, which might otherwise remain undiscovered [33–35]. Based on these general assumptions, the MS-TMT proteomic analyses performed in this study did not assume any specific change in terms of either increased or decreased protein expression levels as direct outcome of sLDR effects to the normal swine brain tissue, and to the Hip region in particular. This methodological approach was also intended to be a robust instrument of analysis for the qualitative and quantitative support of our main working hypothesis, which postulates the occurrence of long-term brain-region based molecular effects after a sLDR and that some of these sLDR-induced effects could be beneficially used to alter neurodegenerative processes. At large, our working hypothesis aims to contribute to a more systematic neurobiology-based background of LDR therapeutic effects for future neuroradiotherapeutic procedures able to contrast, halt, or at least delay, the natural progression and clinical manifestations of neurodegenerative processes, and possibly also other non-neurodegenerative brain disorders such as psychiatric disorders [36]. In support of our hypothesis, recent meta-analyses data revealed the absence of significant dose-effect correlations between ionizing radiation exposure during adulthood and the risk of brain tumors or long-term cognitive deficits in elderly atomic bomb survivors [37, 38]. These latter findings look to confirm and foresee the possibility to modulate either neurological or psychiatric manifestations, or both, by calibrating and adjusting dosage, rate, and precise neuroanatomical localization for the LDR exposure as the specific characteristics of the disease and patient features would indicate. For example, while atomic bomb survivors (young adult individuals at the time of the exposure) fall on the LDR side of the radiation exposure spectrum, specific populations exposed to higher doses of radiation, such as clean-up workers of nuclear power plant accidents (young adult individuals at the time of the exposure as well), have been found at higher risk of severe consequences [39]. These evidence-based findings seem to confirm in general, once again, the validity of the hormetic hypothesis effect of radiation, especially at low doses or rates [40].

In this study, the proteomic results showed detectable expression level changes for a total of 310 proteins in the Hip of RAD vs. SH brains, and based on more stringent criteria (see Methods), *TPM1*, *TPM2*, *TPM3* and *TPM4* expression level changes were found to be particularly more abundant in the Hip of RAD vs. SH group. Moreover, these increased levels, specifically for *TPM1* and *TPM4*, were confirmed by WB analyses.

Most of the Tropomyosins (TPMs) and their multiple isoforms are characteristically located in the cytoplasm of non-muscle cells, that is, neuronal and non-neuronal cells [41]. TPMs isoforms have been mostly implicated in actin interactions, including regulation of filopodia formation, cytoskeletal stabilization, neuronal morphogenesis, rescuing of cell transformation, support of intracellular transport and regulation of non-muscle myosin 2A (*NM2A* activity) [41]. These results, while novel, are also of particular interest since non-muscle myosin proteins, such as *NM2A*, have been shown to produce different TPM-associated effects in powering cytoskeletal contractions, and have been described as altered in several neurodegenerative processes including AD, PD, ALS and MS [42–44]. Furthermore, TPMs have been reported to be involved in various neurodevelopmental aspects [45].

Interestingly, non-muscle myosin proteins have been demonstrated to interfere with Tau phosphorylation mechanisms [46]. Very intriguingly, this last aspect would fit well with some previous findings showing that one of the sLDR-induced effects is actually on pTau expression levels in different regions of the brain [22]. Furthermore, in the CNS, TPMs play a functional role for neurite outgrowth processes, including branching and synapse formation mechanisms [47]. More specifically, pre-synaptic *TPM1* and post-synaptic *TPM4* are important for synaptic function, structural organization, and neurotransmitter activities [47, 48]. Moreover, *TPM4* plays a critical and specific role in neurite branching [49]. Altogether, these TMPs-related functions, as well as their modulators Tmod1 and Tmod2 [50], interact with actin-cytoskeleton-related neurodegeneration processes (i.e., Tau phosphorylation), and largely substantiate the likelihood that a sLDR could beneficially modulate those processes sustaining synaptic strength, synaptic stability, and neuroplasticity in general. For example, TPMs have been shown to influence specific aspects of neuronal size and shape [51]. While some studies suggested that the dysregulation of TPMs is a component of brain injury and neurodegeneration, TPMs’ crucial activities at the synapse level may also indicate a direct role as potential mediators for important neuronal repair mechanisms [47].

Beyond cyto-structural protein changes, and based on these proteomic-WB verified results, we observed increased expression levels of *NPY* and *PCP4* in the Hip of RAD vs. SH swine. *NPY* expression levels have been shown to be reduced in AD-linked processes, and *NPY* fragments have been found to be neuroprotective in a mouse model of AD by ameliorating neurodegenerative pathology [52–54]. More specifically, *NPY* appears to be an anti-inflammatory molecule, and evidence suggests that the modulation of *NPY* expression levels in neurodegenerative disorders actually may be associated with endogenous neuroprotective mechanisms [55, 56]. Also, NPY has been shown to promote cell proliferation and protect against inflammation-induced apoptosis [57]. Furthermore, *NPY* has been found to be a regulator of sleep through noradrenergic signaling modulation, which is particularly interesting considering the associations of sleep disruption with the development of dementia [58, 59].

As for *PCP4*, this molecule modulates calcium binding with calmodulin, thus regulating calmodulin activity [60]. Also, it has been shown that *PCP4* positively regulates neurotransmitter release and neurite outgrowth [61]. *PCP4* expression is significantly altered in a region-specific manner in AD and Huntington’s disease (HD) patient brains, potentially contributing to neurodegeneration through the disruption of calmodulin signaling [62]. *PCP4* is thought to be a neuroprotective molecule, playing a major role in the inhibition of apoptosis and neuronal cell death [63], and promotes cellular homeostasis [60]. In addition, *PCP4* has been known to play a role in cerebellar synaptic plasticity as well as spatial and locomotor learning [64, 65]. Finally, the increase of *NPY* and *PCP4* in the Hip of RAD vs. SH larger mammals 28 days after a sLDR exposure suggests a possible lasting neuromodulatory activity globally promoting a positive signaling cascade in contrast to some of the typical processes occurring during neurodegeneration.

In the context of protein-protein interactions based on STRING network analyses, it is evident that many of the brain proteins responding to sLDR exposure fall within the wider network of neurodegenerative interactions. This is not particularly surprising since our MS-identified protein targets are largely involved in synaptic stabilization and signaling. In particular, the detection of *BCL2L1*, a potent inhibitor of cell death [66], within this protein-protein interaction network, is especially interesting, since the proteomic data showed an increased abundance of this molecule in the swine Hip after sLDR. Moreover, this last finding is consistent with the DAVID enrichment analysis, too. In fact, the DAVID analysis identified a significant enrichment of biological processes relating to regulation of apoptosis and neuron death such as *BAX*, *CYCS*, *SARM1*, *NPY*, *STAT1*, *CHMP4B*, *PEA15* and *ANP32A* (**see DAVID data in** S5 Table). Additionally, STRING analysis identified *GUK1* as an interactor with *APRT* within the network of our target proteins, and our proteomic data showed that *GUK1* is significantly decreased in its abundance (down-regulated) after sLDR. Specifically, *GUK1* is an enzyme responsible for recycling GMP and has been indicated to play a role in tumor growth and survival [67]. Consequently, the decreases of both *APRT* and *GUK1*, as identified by this MS-proteomic analysis, suggest an alteration in phosphate salvage and recycling, possibly with a positive impact on cell metabolism and mitochondrial function. This further observation is again coherent with the DAVID analysis, which identified several mitochondrial cell components and metabolic-related biological functions as enriched in those proteins differentially abundant in the swine Hip after a sLDR in comparison to SH Hip samples.

It is important to emphasize here that we did not detect any compelling evidence that a total-body sLDR exposure to normal swine caused massive proteomic changes after a period of 28 days. Indeed, this last possible occurrence would have suggested a more global and likely detrimental impact on cognitive functioning associated to the Hip region. Rather, our data suggest the activation of possible compensatory or adaptive mechanisms in response to sLDR, which may beneficially bolster synaptic stability and neuroplasticity, together with neuroprotective and anti-inflammatory signaling cascade processes. Intriguingly, very recent proteomic analyses examining brain tissues from AD resilient cases revealed that they are indeed characterized by the enrichment of actin filament-based processes [68]. These recent findings would represent a coherent outcome in relationship to the increased levels of TPMs identified in our study, and would further support the potential interventional use of sLDR as a therapeutic tool in order to increase the resilience of the brain to AD pathology and even providing a positive effect on specific cognitive skills or cognition in general [69].

Based on our proteomic WB-verified data, future larger dose-effect investigations to verify and specify the modulatory, possibly beneficial, effects of LDR on mitochondria, nucleosome, cellular metabolism and calcium signaling pathways are warranted. Of course, these studies should be extended to brain regions beyond the Hip. In general, our new findings seem to also ultimately promote the methodological merit for a region-specific approach to WB verification of proteomic results in an unbiased manner but still in a highly targeted way to better understand the nuances of the neuromolecular changes affecting the brain after a sLDR exposure on the Hip, and probably on other regions of the brain and CNS in general.

While our study provides new molecular data and novel views on the use of sLDR, it has some limitations. In fact, we did not assess the long-term cognitive and non-cognitive effects in our experimental animals, and if these effects would have been present and identified, they could have been used to establish the neurological and behavioral phenotypes of this sLDR exposure. Also, we used a total-body radiation approach for this experiment. However, we do not know if a cranial or brain-region focused sLDR exposure could yield similar or even better results. Finally, we did not use a specific human-disease animal model, that is, including other possible genetic factors influencing the pathogenesis of a specific disease (for example, the *APOE4* allele for AD), to fully confirm that sLDR could have a beneficial effect in terms of neurodegeneration signals. All these aspects, though, will be necessarily part of near future and exciting studies.

## Materials and methods

Animal handling procedures were performed as described previously in compliance with ARRIVE (https://arriveguidelines.org/arrive-guidelines) and the National Research Council for the ethical handling of laboratory animal guidelines [22]. The protocol was approved by the Institutional Animal Care and Use Committee of Uniformed Services University (protocol #PHA-18-942), and all efforts were made to minimize animal suffering. Six-month-old male Gottingen minipigs were purchased from Marshall Farms Group Ltd. USA (North Rose, NY, USA) and ~2 weeks after arrival to the animal facility they received 1.79 Gy of bilateral total-body Cobalt (^60^Co) radiation (RAD group, n = 9) under deep anesthesia (0.485–0.502 Gy/min dose rate; 4.4 mg/kg-2 mg/kg Ketamine/Xylazine) as described in Iacono et al. (2021) [22]. SH animals (n = 6) received anesthesia but were not transported to the radiation facility. Any animals exhibiting signs of radiation injuries were euthanized prior to the study end point based on approved criteria for early euthanasia, including severe lethargy. All animals included in the study analyses demonstrated no overt signs of neurological or systemic illness, as assessed by veterinary staff. There were no significant differences in body weight across the study time course between SH and RAD animals.

### Tissue sample preparation

As earlier described [22], animals were euthanized by intracardial injection of Euthasol (4.5 ml/kg) after 28 days recovery and the brains were dissected out from the skull. The left hemisphere was flash-frozen in chilled liquid isopentane on dry ice for molecular analyses, kept at -80°C until use. Frozen left-brain hemispheres were sectioned on a cryostat at -20°C in 100μm thick coronal sections microdissected into the hippocampus (Hippo) as well as the frontal cortex, cerebellum and other regions (not discussed here). Dissections followed the Gottingen Minipig Brain Atlas (https://www.cense.dk/miniswine_atlas) anatomical delineations.

Hip tissue from each animal (~600-900mg per animal) were homogenized for sample preparation as described before [22], with total protein content determination obtained using the Micro BCA assay (Thermo-Fisher Scientific, 23235, Waltham, MA, USA). Briefly, hippocampus tissue from each biological replicate was homogenized in glass dounce homogenizers with ice cold lysis buffer, centrifuged, and supernatant collected, aliquoted and frozen. Samples were used for MS-based proteomic and Western blot (WB) analyses.

### Tandem Mass Tag (TMT) proteomics procedures and data analysis

#### Isobaric mass tag labeling and fractionation

Proteins (50 ug) were reduced with 50mM Dithiothreitol in 10 mM Triethylammonium bicarbonate (TEAB) at 60°C for 45 minutes followed by alkylating with 100 mM Iodoacetamide in 10 mM TEAB at room temperature in the dark for 15 minutes. MS interfering reagents were removed by precipitating proteins by adding 8 volumes of 10% trichloroacetic acid in cold acetone at -20°C for 2 hours, then centrifuged at 16,000 g for 10 minutes at 4°C. The protein pellet was washed twice with an equivalent 8 volumes cold acetone and centrifuged at 16,000 g for 10 minutes at 4°C. Each of the 12 protein pellets (50 ug) were resuspended and digested overnight at 37°C in 100 uL 100 mM TEAB with 5 ug Trypsin/Lys-C per sample and labeled with a unique TMTpro 16-plex reagent (Thermo Fisher, LOT # VJ313476) according to the manufacturer’s instructions. All 12 TMT labeled peptide samples were combined, dried by vacuum centrifugation, resuspended in 100 μL 200mM TEAB buffer and filtered through Pierce Detergent removal columns (Fisher Scientific PN 87777) to remove excess TMT label, small molecules and lipids. Peptides in the flow through were diluted to 2 mL in 10 mM TEAB and fractionation on a XBridge C18 Column (5 μm, 2.1 x 100 mm column (Waters) using a 0 to 90% acetonitrile in 10 mM TEAB gradient over 85 min at 250 μL/min on an Agilent 1200 series capillary HPLC with a micro-fraction collector. Eighty-four 250 ul fractions were collected and concatenated into 24 fractions according to Wang et al. (2011) [70] and dried by vacuum centrifugation.

#### Mass spectrometry analysis

Peptides in each of the 24 fractions were analyzed on an Orbitrap-Fusion Lumos (Thermo Fisher Scientific) interfaced with an Easy-nLC1100 UPLC by reverse-phase chromatography on a 75 μm x 20 cm picofrit column (New Objective Littleton, MA) in house packed with ReproSIL-Pur-120-C18-AQ 3 μm, 120 Å (Dr. Albin Maisch, Germany), using a 2%–90% acetonitrile in 0.1% formic acid gradient over 110 min at 300nl/min. Eluting peptides were sprayed into the mass spectrometer through a 1 μm emitter tip (New Objective) at 2.3 kV. Survey scans (MS) of precursor ions were acquired from 375–1500 m/z at 120,000 resolution at 200 m/z with automatic gain control (AGC) at 4e5 and a 50 ms maximum injection time. Precursor ions were individually isolated within 0.7 m/z by data dependent monitoring and 15s dynamic exclusion, and fragmented using an HCD activation collision energy 35. Fragmentation spectra (MS/MS) were acquired using a 1.25e5 AGC and 86 ms maximum injection time (IT) at 50,000 resolution.

#### Data analysis

Fragmentation spectra were processed by Proteome Discoverer v2.4 (PD2.4, ThermoFisher Scientific) and searched with Mascot v.2.8.0 (Matrix Science, London, UK) against RefSeq2021_Sus database. Search criteria included trypsin enzyme, one missed cleavage, 3 ppm precursor mass tolerance, 0.01 Da fragment mass tolerance, with TMTpro on N-terminus and carbamidomethylation on C as fixed and TMTpro on K, oxidation on M, deamidation on N or Q as variable modifications. Peptide identifications from the Mascot searches were processed within PD2.4 using Percolator at a 5% False Discovery Rate confidence threshold, based on an auto-concatenated decoy database search. Peptide spectral matches (PSMs) were filtered for Isolation Interference <30%. Relative protein abundances of identified proteins were determined in PD2.4 from the normalized median ratio of TMT reporter ions, having signal to noise ratios >4, from all PSMs from the same protein. Technical variation in ratios from our mass spectrometry analysis is less than 10% [71].

### Western Blot (WB) procedures

For each sample, enough protein for 2 μg/μl concentration was mixed with dH2O and by volume 25% 4x Laemmli Sample Buffer (Bio-Rad Laboratories, Inc., 1610747, Hercules, CA, USA) and 10% 10x NuPAGE Sample Reducing Agent (Life Technologies, 2353153, Carlsbad, CA, USA), then denatured for 10 min at 70°C before electrophoresis. 10ug of protein sample was loaded into each well of Novex Nupage 4–12% Bis-Tris Gels (Life Technologies, NP0329, Carlsbad, CA, USA) and electrophoresed at constant 200 V for 30 min (except for *PCP4*, which was run on Novex 10–20% Tricine Gels for low molecular weight proteins and electrophoresed at constant 180 V for 60 min). The protein ladder well was loaded with 2.5ug Precision Plus Protein Dual Color Standards (Bio-Rad Laboratories, 1610374, Hercules, CA, USA). The iBlot2 dry transfer method (Life Technologies, IB21001, Carlsbad, CA, USA) was utilized to transfer gels onto PVDF membranes. These were incubated for 10min at RT with Ponceau-S Staining Solution (Boston BioProducts, ST-180, Ashland, MA, USA), rinsed with dH2O and scanned on an Epson Perfection V39 (Epson America, Inc., Los Alamitos, CA, USA) in 8-bit gray scale at 300dpi for whole protein visualization. Then membranes were rinsed in 1x TBST and blocked for 1h at room temperature in 5% milk in 1x TBST. Membranes were incubated overnight at 4°C in primary antibody solutions (appropriate working concentrations of primary antibodies (*see below*) were diluted in the 5% milk in 1x TBST, except for *NPY* which was diluted in 5% BSA in 1x TBST and incubated for 3 days). After rinsing the membranes 3x 5 min in TBST, they were incubated for 2h at RT in appropriate HRP tagged secondary antibodies (see below) diluted 1:2000 in the same kind of buffer used for primary incubation. Membranes were rinsed in 1x TBST for 3x 5min and in 1x TBS for 1x 5min, then incubated for 1 min in chemiluminescent substrate (SuperSignal West Pico Chemiluminescent Substrate, Thermo-Fisher Scientific, 34577, Waltham, MA, USA) and imaged on LiCor C-Digit Blot Scanner (LiCor Biosciences, Lincoln, NE, USA). Densitometry was performed using Fiji ImageJ software v2.9.0 (NIH, Bethesda, MD, USA), with all protein intensities normalized to total protein signal intensity.

#### Primary antibodies

To verify the proteins identified by the MS-proteomic analysis as being differentially abundant after sLDR with a high likelihood of coherent verification through MS and WBs results, we measured the expression level changes only of those proteins within the following parameters: significant range (*p*<0.05), log2 fold change (FC) >1, and high confidence (see S2 Table). Based on these stringent parameters, we selected the following eight target proteins:

- *Tropomyosins (TPMs) 1–4* (*TPM1*, 1:400, Invitrogen, Thermo-Fisher Scientific, PA5-29846, Waltham, MA, USA; *TPM2*, 1:1500, Proteintech, 11038-1-AP, Rosemont, IL, USA; *TPM3*, 1:2500, Invitrogen, Thermo-Fisher Scientific, PA5-52644, Waltham, MA, USA; *TPM4*, 1:3000, Proteintech, 67244-1-Ig, Rosemont, IL, USA). As a reminder, all these TPMs proteins are involved in actin-myosin interactions and recently were demonstrated to also interact with non-muscle myosin molecules (including neurons) and as such are involved in different physiological and pathological processes [41];
- *Purkinje Cell Protein 4* (*PCP4*, 1:1500, Proteintech, 14705-1-AP, Rosemont, IL, USA), a protein involved in calcium signaling and synaptic plasticity (also known as *PEP19*);
- *Neuropeptide Y* (*NPY*, 1:500, Cell Signaling Technology, 11976, Danvers, MA, USA), a protein associated with stress resilience and autonomic functioning;
- *Sorbin and SH3 domain containing 1* (*SORBS1*, 1:350, Proteintech, 13854-1-AP, Rosemont, IL, USA), a regulator of cell adhesion and involved in facilitating insulin-mediated glucose transport;
- *Adenine Phosphoribosyltransferase* (*APRT*, 1:500, Invitrogen, Thermo-Fisher Scientific, PA5-76741, Waltham, MA, USA), involved in the nucleotide salvage pathway.

Specifically, in terms of up- and down-regulation, *TPM1*, *TPM2*, *TPM3*, *TPM4*, *PCP4*, and *NPY* expression levels were increased and *APRT* and *SORBS1* expression levels were decreased in the Hip proteomic profiles of RAD vs. SH animals (see Results).

#### Secondary antibodies

HRP tagged secondary antibodies goat anti-mouse and goat anti-rabbit (ab97040 and ab97080, respectively, 1:2000, Abcam, Cambridge, MA, USA) were used for chemiluminescent detection of protein signal.

### Statistical analysis

For the MS-proteomics data analyses and methods see paragraph Tandem Mass Tag (TMT) proteomics procedures and data analysis. P-values were calculated using *t*-test for individual proteins with biological replicates, because there are only two conditions (RAD vs SH). Grouped abundances CV = 100 × std. dev/median. Z-score transformation of normalized protein abundances from a quantitative proteomics analysis using isobaric mass tags was applied before performing the hierarchical clustering based on Euclidean distance and complete (furthest neighbors) linkage.

Densitometry data from WB were obtained by imageJ and analyzed by 1-tailed, unpaired *t*-tests using GraphPad Prism v9.4.1 (La Jolla, CA, USA), with a threshold of *p*<0.05 used to determine significant differences. All WB experiments were performed in technical triplicate and target/whole protein relative density ratios were averaged between the same samples for analysis.

## Supporting information

S1 Fig Preliminary checks for following proteomic analyses in RAD vs. SH swine hippocampus. (A) Protein abundances of each hippocampus sample were determined to be sufficient for proteomic analysis. (B) 2D coronal section image to visualize anatomical markers for dissection accuracy of Hip from Göttingen mini pig (https://cense.au.dk/fileadmin/minipig/atlas/index.html) [29]; reprinted from *Orlowski D*, *Glud AN*, *Palomero-Gallagher N*, *Sørensen JCH & Bjarkam CR*. *Online histological atlas of the Göttingen minipig brain*. *Heliyon 5 (2019) e01363*. *Doi*: 10.1016/j.heliyon.2019.e01363 under a CC BY license, with permission from Dr. Orlowski, original copyright 2016.
(PDF)

S2 Fig Representative full blot for total protein and target protein staining. For Western blotting, 10 μg of protein was added per well for each sample. For all eight target proteins that underwent WB testing, we show a representative blot with Ponceau S staining for total protein (left) and subsequent target protein staining (right). The band that was used for quantification is indicated by an arrow with approximate molecular weight. APRT (A), SORBS1 (B), TPM1 (C), TPM2 (D), TPM3 (E), TPM4 (F), PCP4 (G) and NPY (H). Quantification results are shown in Fig 3a.
(PDF)

S1 Table All proteins. Proteomic data of all proteins identified through MS.
(XLSX)

S2 Table Increased and decreased proteins. Proteomic MS data of significantly up and down regulated hippocampal proteins after LDR exposure.
(XLSX)

S3 Table STRING analysis. STRING protein interactions and enrichment analysis of the network involving 8 target proteins (TPM1, TPM2, TPM3, TPM4, NPY, PCP4, APRT and SORBS1) (Relates to Fig 3c).
(XLSX)

S4 Table STRING analysis with neurodegenerative inputs. STRING protein interactions and enrichment analysis of 8 target proteins within previously studied neurodegenerative network (Relates to Fig 4b).
(XLSX)

S5 Table DAVID analysis. DAVID enrichment analysis of differentially abundant proteins after low dose radiation (Relates to Fig 5).
(XLSX)
