# Supplementary material for: Proteomic changes in the hippocampus of large mammals after total-body low dose radiation
Source: PLoS One. 2024 Mar 1;19(3):e0296903. doi: 10.1371/journal.pone.0296903 (PMC10906861; doi:10.1371/journal.pone.0296903)
Supplement: S1 Fig — (A) Protein abundances of each hippocampus sample were determined to be sufficient for proteomic analysis. (B) 2D coronal section image to visualize anatomical markers for dissection accuracy of Hip from Göttingen mini pig (https://cense.au.dk/fileadmin/minipig/atlas/index.html) [29]; reprinted from Orlowski D, Glud AN, Palomero-Gallagher N, Sørensen JCH & Bjarkam CR. Online histological atlas of the Göttingen minipig brain. Heliyon 5 (2019) e01363. Doi: 10.1016/j.heliyon.2019.e01363 under a CC BY license, with permission from Dr. Orlowski, original copyright 2016. (PDF) [file pone.0296903.s001.pdf]

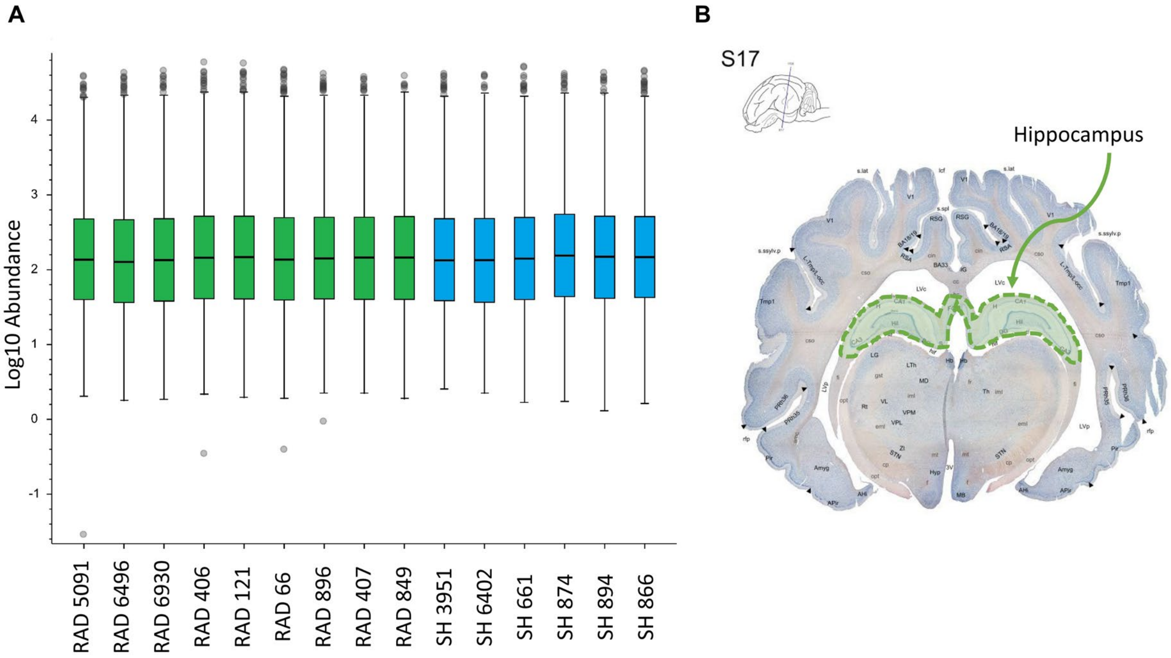

S1 Fig. Preliminary checks for following proteomic analyses in RAD vs. SH swine Hippocampus. (A) Protein abundances of each hippocampus sample were determined to be sufficient for proteomic analysis. (B) 2D coronal section image to visualize anatomical markers for dissection accuracy of Hip from Göttingen mini pig (https://cense.au.dk/fileadmin/minipig/atlas/index.html) (29); reprinted from *Orlowski D, Glud AN, Palomero-Gallagher N, Sørensen JCH & Bjarkam CR. Online histological atlas of the Göttingen minipig brain. Heliyon 5 (2019) e01363. Doi: 10.1016/j.heliyon.2019.e01363* under a CC BY license, with permission from Dr. Orlowski, original copyright 2016.
