# Supplementary material for: Proteomic changes in the hippocampus of large mammals after total-body low dose radiation
Source: PLoS One. 2024 Mar 1;19(3):e0296903. doi: 10.1371/journal.pone.0296903 (PMC10906861; doi:10.1371/journal.pone.0296903)
Supplement: S2 Fig — For Western blotting, 10 μg of protein was added per well for each sample. For all eight target proteins that underwent WB testing, we show a representative blot with Ponceau S staining for total protein (left) and subsequent target protein staining (right). The band that was used for quantification is indicated by an arrow with approximate molecular weight. APRT (A), SORBS1 (B), TPM1 (C), TPM2 (D), TPM3 (E), TPM4 (F), PCP4 (G) and NPY (H). Quantification results are shown in Fig 3a. (PDF) [file pone.0296903.s002.pdf]

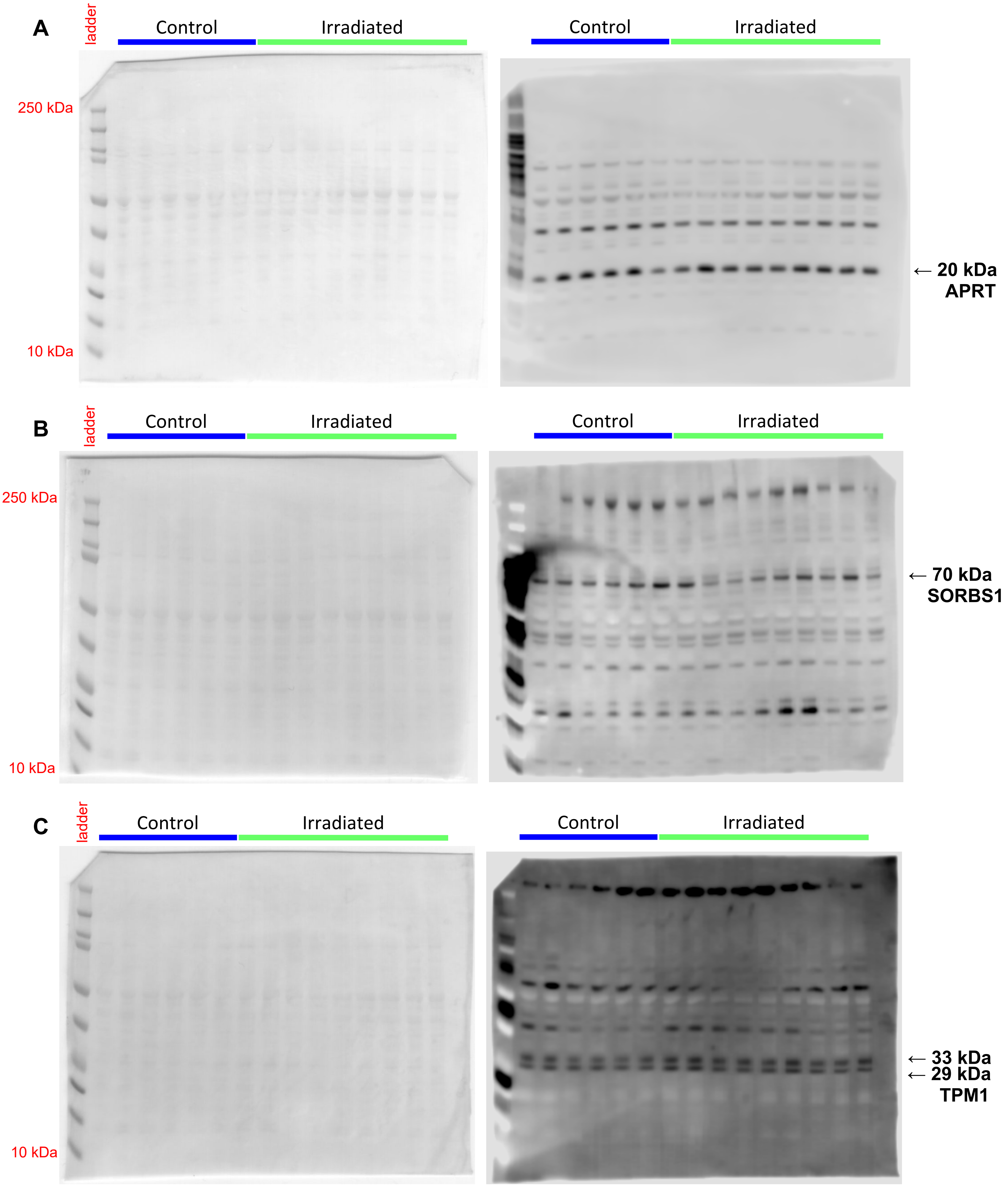
A
ladder
Control
Irradiated
250 kDa
10 kDa
Control
Irradiated
← 20 kDa
APRT
B
ladder
Control
Irradiated
250 kDa
10 kDa
Control
Irradiated
← 70 kDa
SORBS1
C
ladder
Control
Irradiated
10 kDa
Control
Irradiated
← 33 kDa
← 29 kDa
TPM1

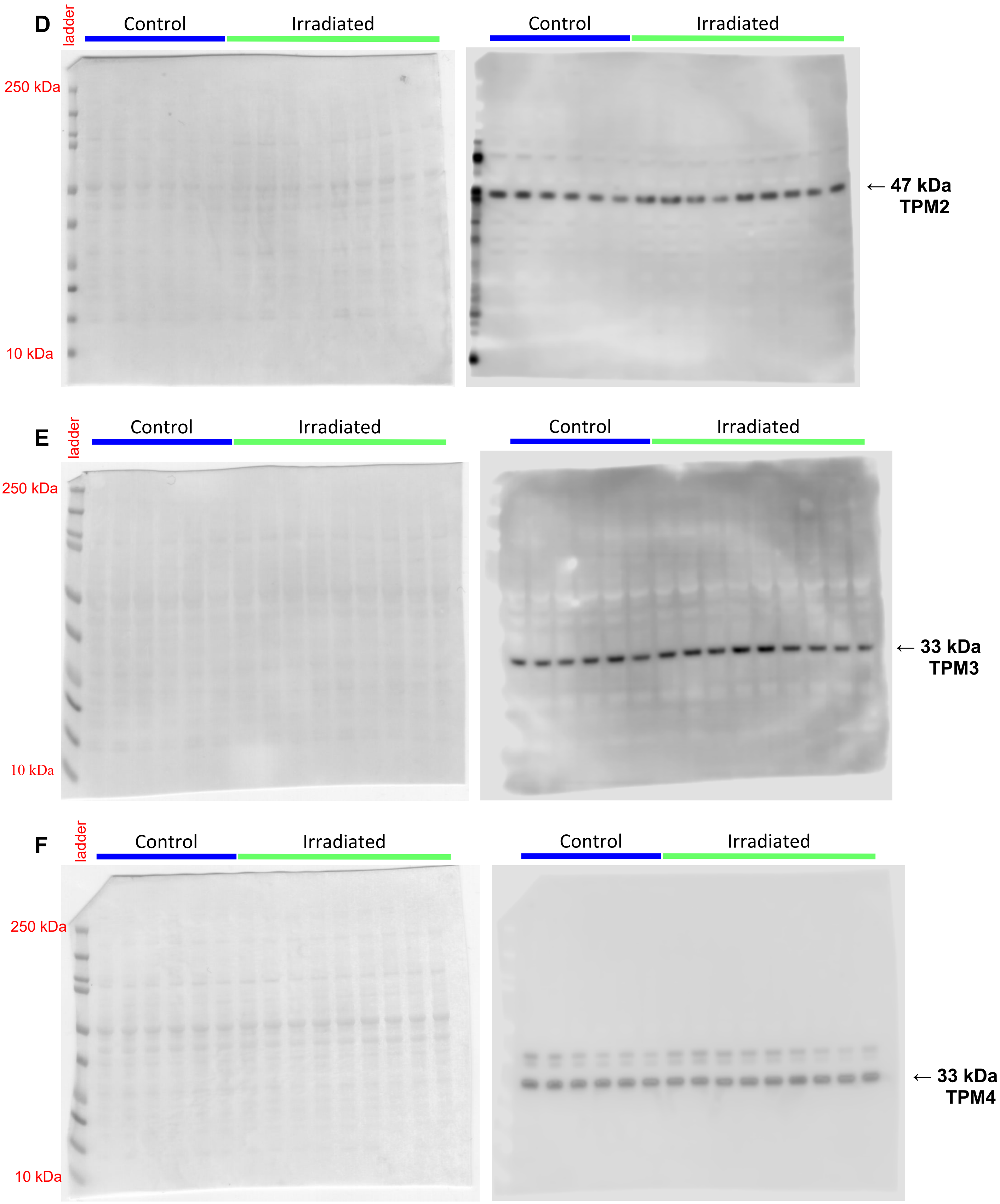
D
ladder
Control
Irradiated
250 kDa
10 kDa
Control
Irradiated
← 47 kDa
TPM2
E
ladder
Control
Irradiated
250 kDa
10 kDa
Control
Irradiated
← 33 kDa
TPM3
F
ladder
Control
Irradiated
250 kDa
10 kDa
Control
Irradiated
← 33 kDa
TPM4

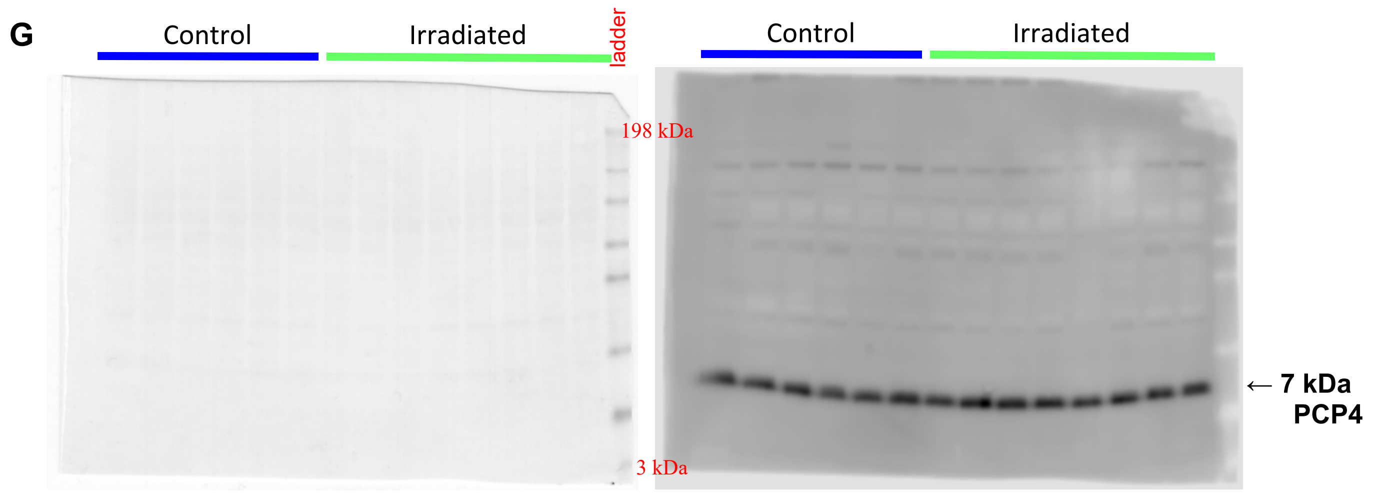

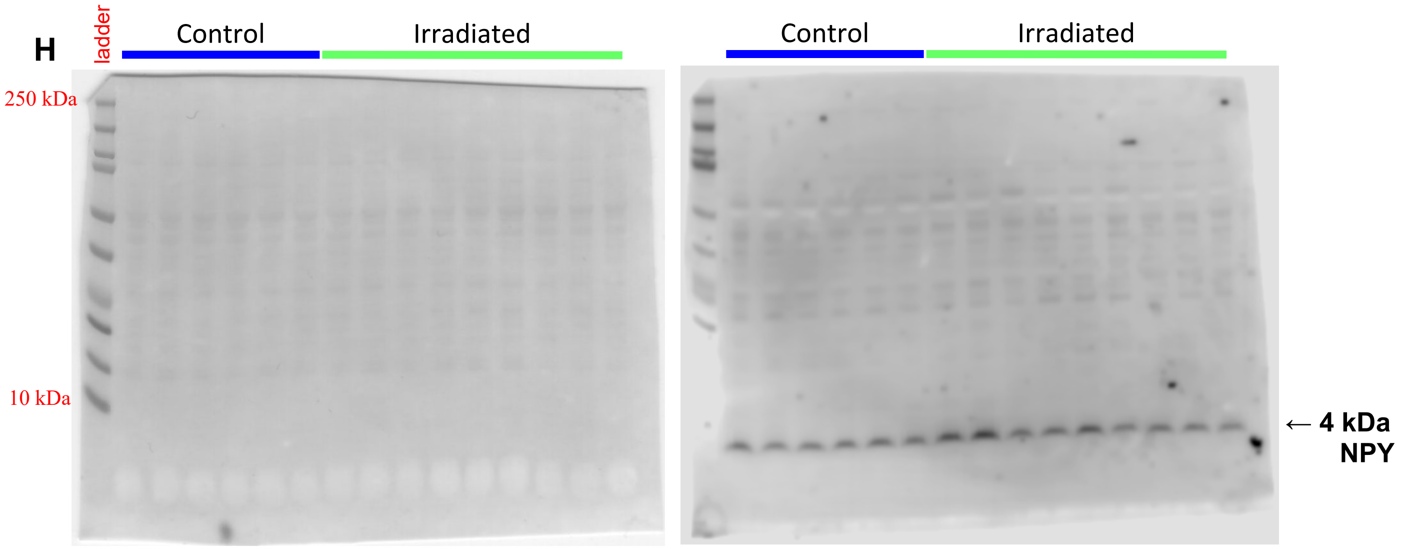

S2 Fig. Representative full blot for total protein and target protein staining. For Western blotting, 10 µg of protein was added per well for each sample. For all eight target proteins that underwent WB testing, we show a representative blot with Ponceau S staining for total protein (left) and subsequent target protein staining (right). The band that was used for quantification is indicated by an arrow with approximate molecular weight. APRT (A), SORBS1 (B), TPM1 (C), TPM2 (D), TPM3 (E), TPM4 (F), PCP4 (G) and NPY (H). Quantification results are shown in Fig. 4a.
